# Peri‐ictal psychiatric manifestations in people with epilepsy: An umbrella review

**DOI:** 10.1002/epi4.12949

**Published:** 2024-05-30

**Authors:** Carlos Alva‐Diaz, Miguel Cabanillas‐Lazo, Alba Navarro‐Flores, Raisa N. Martinez‐Rivera, Maria Valdeiglesias‐Abarca, Krystel Acevedo‐Marino, Kevin Pacheco‐Barrios, Ramiro Ruiz‐Garcia, Jorge Burneo

**Affiliations:** ^1^ Grupo de Investigación Neurociencia, Efectividad Clínica y Salud Pública Universidad Científica del Sur Lima Peru; ^2^ Servicio de Neurología, Departamento de Medicina y Oficina de Apoyo a la Docencia e Investigación (OADI) Hospital Daniel Alcides Carrión Callao Peru; ^3^ Universidad de Huánuco Huánuco Perú; ^4^ International Max Planck Research School for Translational Psychiatry (IMPRS‐TP) Munich Germany; ^5^ Red de Eficacia Clinica y Sanitaria (REDECS) Lima Peru; ^6^ Universidad Nacional de Piura Piura Peru; ^7^ Universidad Ricardo Palma Lima Peru; ^8^ Neuromodulation Center and Center for Clinical Research Learning Spaulding Rehabilitation Hospital and Massachusetts General Hospital, Harvard Medical School Boston Massachusetts USA; ^9^ Vicerrectorado de Investigación, Unidad de Investigación Para la Generación y Síntesis de Evidencias en Salud Universidad San Ignacio de Loyola Lima Peru; ^10^ Department of Neuropsychiatry National Institute of Neurology and Neurosurgery Mexico City Mexico; ^11^ Epilepsy Program and Neuroepidemiology Unit, Department of Clinical Neurological Sciences, Schulich School of Medicine Western University London Ontario Canada

**Keywords:** diagnostic and statistical manual of mental disorders, epilepsy, mental disorder, prevalence, systematic review

## Abstract

**Objective:**

We aimed to conduct an umbrella review to summarize the existing evidence regarding the prevalence of peri‐ictal psychiatric manifestations (PM) in people with epilepsy (PWE) including pre‐ictal, ictal, and postictal stages.

**Method**s**:**

Databases were searched up to June 2023 for systematic reviews (SR) of observational studies that included patients with epilepsy peri‐ictal PM. Data selection, data extraction, and risk of bias assessment (with the AMSTAR‐2 instrument) were performed by two independent reviewers. We performed a narrative synthesis using previous guidelines. We used a self‐developed decision table according to the GRADE system adapted for narrative outcomes if the certainty of outcomes was not determined by systematic review authors.

**Results:**

Four SRs were included comprising 66 primary studies (*n* = 10 217). Three SRs evaluated one period (pre‐ictal, ictal, and postictal), and one did not determine it. During the pre‐ictal period, the more prevalent symptom was confusion, although with a low certainty (due to the heterogeneity and serious risk of bias). One systematic review that only included case reports evaluated the ictal period, finding mood/anxiety disorders, psychosis, and personality changes. The postictal period included the most PM (anxiety: 45.0% and depressive symptoms: 43.0%), with very low certainty, due to risk of bias, potential publication bias, heterogeneity, and failure to report the confidence intervals.

**Significance:**

With very low certainty, epileptic periods are characterized by a wide spectrum of PM, being postictal symptoms the most prevalent, predominantly anxiety, and depressive symptoms. Further understanding of these PM of epilepsy could improve the attention of the people with epilepsy.

**Plain Language Summary:**

In this review of reviews, we summarize the frequency in which psychiatric manifestations occur in relation to an epileptic seizure. A total of 10 217 patients were reported in the reviews. The most common manifestations included symptoms of anxiety and depression, as well as changes in the normal behavior of the patient. These manifestations occurred most frequently right after the seizure finished.

**Key points**

- We conducted an umbrella review summarizing the prevalence of peri‐ictal psychiatric manifestations (PM) in people with epilepsy (PWE).
- We included observational studies assessing PM in PWE and performed a narrative synthesis.
- We included 66 primary studies and 10 217 patients, analyzing the PM in the pre‐ictal, ictal, and postictal period.
- The most common manifestations included symptoms of anxiety, depression, psychosis, personality changes, and confusion.
- The postictal manifestations were the most prevalent, mostly anxiety, and depressive symptoms.

## 1 INTRODUCTION

Epilepsy is one of the most common neurological diseases.^1^ The incidence is higher in men, and in low‐ and middle‐income countries,^2^ that is, being around two‐fold higher in Latin America, generating high burden worldwide.^3^, ^4^, ^5^

It is estimated that approximately 20%–30% of people with epilepsy (PWE) present a psychiatric comorbidity,^6^ which is a higher prevalence than the general population.^7^ Psychiatric manifestations (PM) in PWE exist in different contexts: (1) Interictal PM, which are not temporarily related to seizure activity, (2) Peri‐ictal (pre‐ictal, postictal, and ictal phases) PM, which have a temporal relationship with seizure activity,^8^ (3) PM associated with antiseizure drugs (e.g., psychosis associated to levetiracetam),^9^ and (4) Para‐ictal PM which are clinically known as “alternative psychopathology,” and have a close relationship with “abrupt epilepsy control”—a term known in the field of neurophysiology as “EEG forced normalization”.^10^

Peri‐ictal PM occur in a close temporal context of the epileptic seizure. In generalized epilepsy, these PM can occur in up to 26% of the patients. The most frequently reported are affective (47%) and anxiety/panic symptoms (26%).^11^ In focal epilepsies, up to 60% of the patients present PM,^12^ mostly depressive symptoms (50%), anxiety symptoms (30%), and psychosis (13%)—the latter being higher in patients with temporal lobe epilepsy (16%).^6^ According to subtypes of peri‐ictal PM, pre‐ictal PM have a presentation hours or days before a seizure, typically exhibiting a spontaneous remission hours or days after the seizure.^13^ Postictal PM appear after a seizure and following a symptom‐free (hours‐days) period. This has been often described in PWE with treatment‐resistant features, focal epilepsy, and interictal PM.^9^ Lastly, ictal PM had been identified mainly in focal epilepsies. In temporal lobe epilepsies, common manifestations are present in auras, which could include hallucinations or auditory distortions, vertigo, or visual hallucinations and psychic or experiential phenomena, such as déjà vu, jamais vu, or fear. Another example are the so‐called “gelastic crises,” uncontrollable stereotyped laughter (with or without a feeling of joy),^14^ as well as behavioral alterations that include ictal speech and vocalizations,^15^ or affective behaviors (laughter, crying, or fear). In frontal lobe epilepsies, hypermotor seizures were described as “withdrawal behavior” (walk or run).^16^

Characterization of PM during the peri‐ictal period had been scarcely reported, potentially due to the brief duration of the symptoms. Consequently, further standardization of peri‐ictal PM clinical characteristics and differential diagnostic criteria are lacking. There is no clear consensus regarding semiology or typology, classification, diagnosis, and treatment. These could generate misdiagnosis of PWE, increasing the risk of refractory treatment, and the consequent burden on the health and quality of life of the patients and their families. Therefore, we aim to conduct a systematic review of the existing evidence on the prevalence of psychiatric manifestations in PWE that occur peri‐ictally, classifying them according to the ictal phase (pre‐ictal, ictal, and postictal), and by type of epilepsy.

## 2 METHODS

This umbrella review was reported according to the Preferred Reporting Items for Systematic Reviews and Meta‐Analyses (PRISMA).^17^ The study protocol is published in Figshare.^18^

### 2.1 Data sources

We searched in PubMed, PsycInfo, Web of Science, Epistemonikos, and Scopus until June 2023. We designed a search strategy for PubMed and adapted it for its use in the other databases (Table S1). There were no restrictions on language or publication date.

### 2.2 Eligibility criteria

We included Systematic Reviews (SR) of observational studies that included epileptic patients with peri‐ictal (pre‐ictal, ictal, and postictal) PM. We excluded any primary studies such as clinical trials, observational studies, case reports, studies in non‐humans, conference abstracts, and letters.

### 2.3 Study selection

Results from electronic searches were exported to the online tool Rayyan (https://rayyan.qcri.org/), and duplicated records were removed. Screening of the records was performed by two reviewers (ANF and RMR), and any discrepancies were resolved by consensus or by the third reviewer (CAD). Two reviewers (MCL and RMR) assessed inclusion criteria independently by reading the full texts of the potentially relevant studies selected and discrepancies were resolved according to consensus. The complete list of excluded articles is provided in Table S2.

### 2.4 Outcomes

The primary outcome was prevalence or frequency of psychiatric manifestations preceding the seizure (pre‐ictal), following the seizure (postictal) or as an expression of seizure (ictal) in epileptic patients.^19^ Other outcomes were the type of psychiatric manifestation during the seizure according to categories of Diagnostic and Statistical Manual of Mental disorders, Fifth Edition (DSM‐5) (symptoms of mood disorders, depressive Disorders, bipolar and related disorders, anxiety disorders, personality disorders, schizophrenia spectrum, and other psychotic disorders).

### 2.5 Data extraction

Two authors (RMR and MCL) independently carried out data extraction using a data extraction form, and any disagreements were resolved by consensus and ultimately a third author (CAD). We extracted the following information: title of the study, first author, year of publication, number of participants, number of studies included, description of epilepsy, and psychiatric manifestation.

### 2.6 Methodological quality assessment

The quality of all the SR included was assessed with the A MeaSurement Tool to Assess Systematic Reviews (AMSTAR 2) by two authors (MCL and RMR).^20^ Again, disagreements were resolved by consensus or by the third reviewer (CAD).

The AMSTAR 2 quality assessment tool is a 16 item or domain checklist. Seven of these items are considered critical. Shortcomings in any of the critical domains could affect the overall validity of a review. The domains considered critical are as follows: registration of the protocol before starting the review; conduct of an adequate search of the literature; providing justification for the exclusion of individual studies; satisfactory assessment of risk of bias in the studies included in the review; use of appropriate statistical methods in performing a meta‐analysis; accounting for risk of bias when interpreting the results; and evaluation of the presence and effect of publication bias.^20^

According to the AMSTAR‐2 tool, one “no” answer in non‐critical questions means high confidence in the results; more than one negative answer means moderate confidence; “no” answer in critical questions means low confidence; and more than one answer means critically low confidence.

### 2.7 Data synthesis and certainty of evidence

SR that met the inclusion criteria formed the unit of analysis. Only data available from reviews is presented. Results from reviews were synthesized with a narrative technique. It was performed using previous guidelines based on four steps^21^: (1) developing a theory of how the symptoms could be explained (pathophysiological and clinical plausibility); (2) developing a preliminary list of synthesis categories of psychiatric manifestation according to DSM‐5 and the temporality with respect to the ictal manifestation (pre‐ictal, ictal, or postictal); (3) exploring relationships between symptoms using a summary table^12^, ^22^; and (4) assessing the certainty of body of evidence using Grading of Recommendations, Assessment, Development and Evaluation (GRADE).

When the certainty of outcomes was not determined using the GRADE system by systematic review authors; it was calculated by the current umbrella review authors using GRADE. Only systematic reviews that evaluated in a period of time regarding seizure (peri‐ictal period) were assessed. Systematic reviews that included case reports were excluded from GRADE analysis. There is limited guidance about assessing the certainty of evidence in umbrella reviews,^23^ and some reports of adaptation.^24^, ^25^, ^26^ For this reason, for our umbrella review, we used a self‐developed decision table (Table S3). This could be an important contribution to umbrella reviews about prevalence.

### 2.8 Overlap measurement

The primary studies included in each SR were listed in a table (Table S4). We did not perform this analysis in systematic reviews that included only case reports. Then, overlapping was calculated and reported as percentage according to the formula:

$$\left(\frac{N-b}{\mathrm{bc}-b}\right)\times 100$$

whereby *N* represents the total number of primary studies, *b* is the number of primary studies not including overlapping studies and *c* is the number of SR. We interpreted the results following categories: slight (0%–5%), moderate (6%–10%), high (11%–15%), and very high overlap (>15%).^27^

## 3 RESULTS

### 3.1 Study selection

**FIGURE 1:**
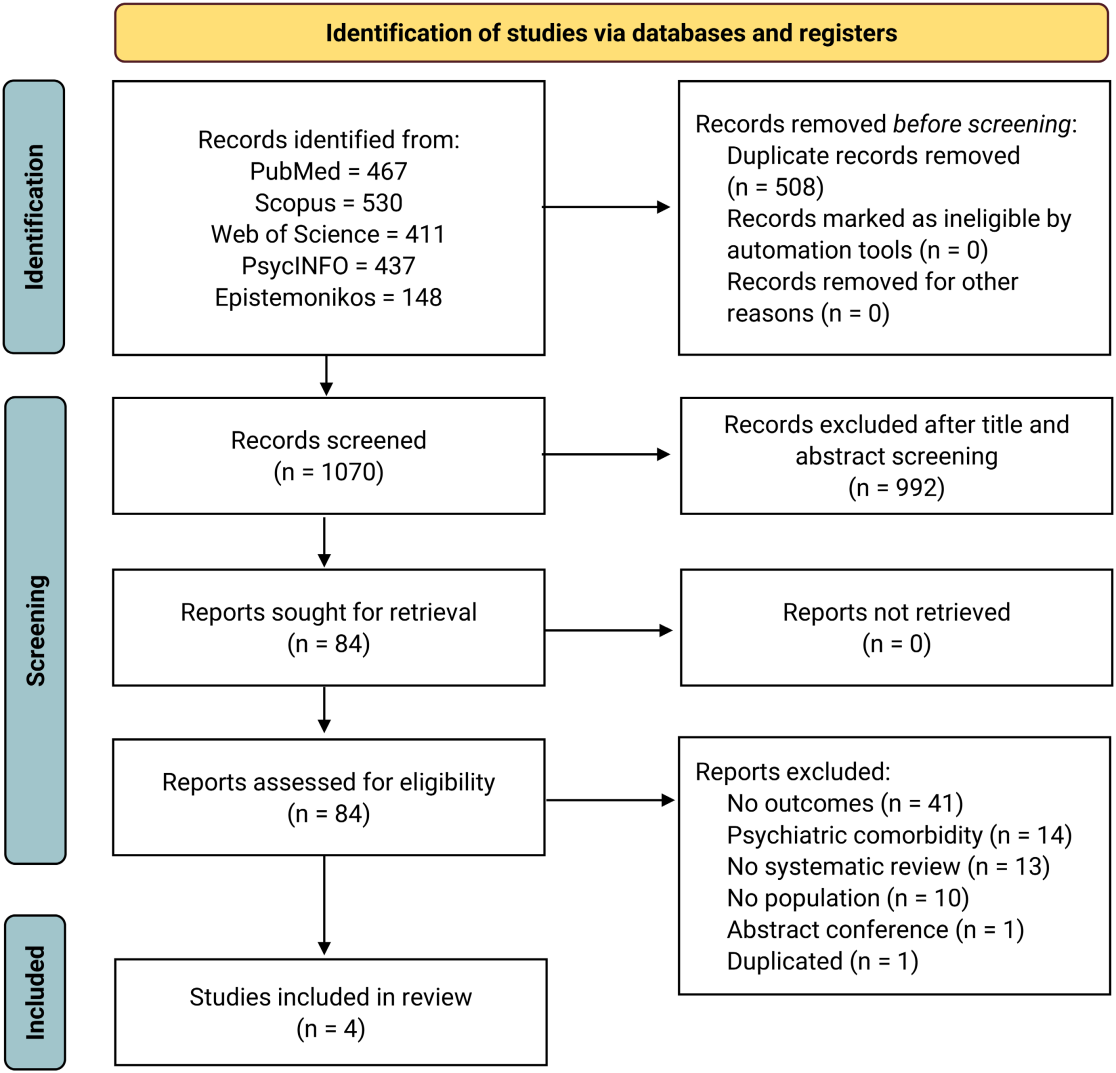
PRISMA flowchart of selected studies.

We identified 1993 studies. After the removal of duplicates, 1070 studies were included for screening. Then, 84 articles were examined by full‐text assessment. Finally, four SRs were included^28^, ^29^, ^30^, ^31^ (Figure 1).

### 3.2 Characteristics of the included studies

Two SR included prevalence studies and two case reports. In total, they included 66 studies. The total number of participants was 10 217, the average age of the patients ranged between 6.1 and 42.8 years. Among studies that report gender, Corbet et al.^28^ proportion of males was 65.1% while Gold et al. included a 62.1% of males. Gold et al.^31^ included studies regarding frontal lobe epilepsy and Corbet et al. included 88.1% of patients with gelastic seizures (Table 1).

**TABLE 1:** Characteristics of systematic reviews included.

Study‐ID	Design	Dead of last search	N included studies	Type of study	N participants	Sex participants	Age participants	Epilepsy description	Risk of bias of included studies	AMSTAR‐2
Corbet (2019)^28^	Systematic review	February 6, 2019	29	Case series	264	Male: 65.1%	9.4 (SD 5.4)	Gelastic seizures were most common (88.1%) Partial or complex partial seizures (40.1%) Generalized tonic–clonic (30.1%)	NR	8/13 (critically low)
Besag (2018)^29^	Systematic review	May, 2017	8	Prevalence studies	1843	NS	NS	NR	NR	2/13 (critically low)
Subota (2019)^30^	Systematic review and meta‐analysis	November 29, 2017	78	Prevalence studies	Range between 26 and 1510	NS	Mean between 6.1 and 42.8	Majority of studies examined people with focal epilepsies	NR	9/16 (critically low)
Gold (2016)^31^	Systematic review and meta‐analysis	December, 2015	35	Case series and report	66	Male: 62.1%	32.7 (SD 24.0)	‐Type of studied epilepsy is Frontal lobe epilepsy. ‐Causes of seizure were known in 53%, with the most common causes being dysplasia and tumor	NR	4/13 (critically low)

### 3.3 Methodological quality assessment

All SR had an AMSTAR‐II score <12 and had critically low confidence. The critical domain regarding the presentation of a list of reasons for the excluded articles was rated negatively in all included systematic reviews (Figure S1 and Table S5).

### 3.4 Data synthesis, certainty of evidence, and overlap measurement

**FIGURE 2:**
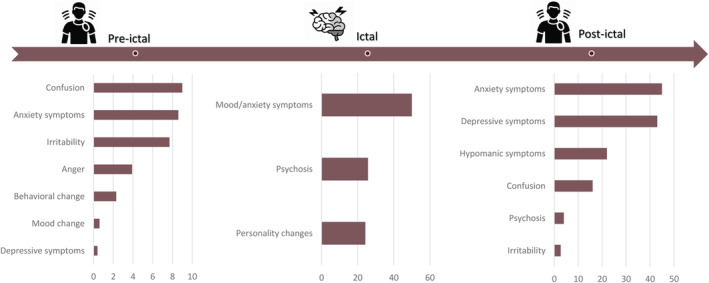
Comparative summary of PMs in PWE. Percentage of patients with psychiatric manifestations according to the pre‐ictal, ictal, and postictal period.

Regarding the pre‐ictal period, the more prevalent two symptoms were confusion and anxiety with 9.0% and 8.6% of prevalence, respectively. Only, data from case reports were reported in the ictal period (mood/anxiety symptoms, psychosis, and personality changes). The postictal period was the period with the most reported psychiatric symptoms (anxiety: 45.0% and depressive symptoms: 43.0%). A summary of PMs is presented in Figure 2 and Table 2.

**TABLE 2:** Prevalence of psychiatric manifestations.

Psychiatric manifestation	Pre‐ictal (%)	Ictal (%)	Postictal (%)	No specified (%)
Anxiety symptoms	Anxiety symptoms: 8.6^a^	Mood/anxiety symptoms^b^	Anxiety symptoms: 45.0^c^	NR
Affective symptoms	Depressive symptoms: 0.4^a^ Irritability: 7.7^a^ Anger: 3.9^a^ Mood change: 0.6^a^	NR	Depressive symptoms: 43.0^c^ Hypomanic symptoms: 22.0^c^ Irritability: 2.7^c^	Rage attacks: 40.6^d^
Psychotic symptoms	NR	Psychosis^b^	Psychosis: 4.0^c^	NR
Behavioral changes	Behavioral change: 2.3^a^ Confusion: 9.0^a^	Personality changes^b^	Confusion: 16.0^c^	NR

*Note*: Prevalence of psychiatric symptoms in epileptic patients according to Diagnostic and Statistical Manual of Mental disorders, Fifth Edition (DSM‐5) and the temporality with respect to the ictal manifestation.

^a^ Obtained from Besag and Vasey.^29^

^b^ Obtained from Gold et al.^31^ based on case reports.

^c^ Obtained from Subota et al.^30^

^d^ Obtained from Corbet et al.^28^

Regarding certainty of the evidence of pre‐ictal psychiatric symptoms,^29^ we started the evaluation from high certainty, because SRs included cross‐sectional studies. We downgraded according to the no performance of the included studies; heterogeneity was not measured, and the confidence interval was not presented. About certainty of the evidence of postictal symptoms,^30^ we started the evaluation with high certainty, because systematic reviews included cross‐sectional studies. We downgraded according to the high risk of bias of 50% of the included studies, and heterogeneity was not presented (Table 3). The certainty of the evidence of ictal symptoms was not assessment^28^ due to the small number of included studies. Furthermore, among the SR analyzed, there was no overlapping (0%) of the primary studies included in each of them.^28^, ^29^, ^30^ Gold et al included only case reports and it was not analyzed.^31^

**TABLE 3:** GRADE summary of findings.

Outcomes	No of participants	No of included studies	Reported prevalence (95% CI)	Quality of the evidence (GRADE)
Pre‐ictal Psychiatric manifestations	1843	8 cross‐sectional studies	Prevalence of individual symptoms was reported^a^	⨁◯◯◯^b^ ^,^ ^c^ ^,^ ^d^ ^,^ ^e^ Very low
Ictal Psychiatric manifestations	59	29 case reports	n/N	NA
Postictal Psychiatric manifestations	Range between 26 and 1510	78 cross‐sectional studies	Prevalence of individual symptoms was reported	⨁⨁◯◯^c^ ^,^ ^f^ Low

Abbreviation: NA, Not Assessment.

^a^ No confidence intervals were presented.

^b^ Risk of bias measurement of individual studies was not performed.

^c^ Heterogeneity was not measured.

^d^ The confidence intervals was not presented.

^e^ Only PubMed was used to select studies.

^f^ Serious risk of bias of individual studies (>25% and <50%).

## 4 DISCUSSION

### 4.1 Summary of findings

Our review found that in the postictal period there was a greater manifestation of psychiatric symptoms, followed by the pre‐ictal and ictal period. Among the most reported symptoms were anxiety and depressive symptoms.

### 4.2 Comparison of our findings with other studies and possible explanatory mechanisms

Regarding peri‐ictal manifestations, anxiety, and depressive symptoms were commonly reported. This finding is comparable to previous reports, although depressive symptoms were reported to be more prevalent than anxiety.^30^ In adults with hypothalamic hamartomas depressive and anxiety symptoms were present in up to 80% of them,^28^ similarly common in PWE with focal seizures originating from the right temporal structures.^32^ Some potential hypotheses regarding the intervening mechanisms that occur during seizures associated with psychiatric manifestations would include the following: (1) Dysregulation of the hypothalamic–pituitary–adrenal axis involved in chronic stress conditions. This would be related to increased epileptogenesis and increased excitability of hippocampal neurons mediated by an excess of related hormones such as corticoliberin^33^, ^35^, ^36^ and (2) the excess of glutamate. This excitatory neurotransmitter has been observed to promote the appearance of depressive symptoms.^34^ The subjective experience described in seizures may influence peri‐ictal symptoms, particularly in focal epilepsies, as well as the awareness of these abnormal sensations, may cause emotional distress. This is likely related to patients' previous psychological and social factors.^32^ The subjective experience of seizures influences peri‐ictal symptoms, particularly in focal epilepsies and the awareness of these abnormal events could be associated with ictal emotional distress which depends on the patients' previous psychological and social factors.^35^, ^36^

In the pre‐ictal phase, the most prevalent symptoms were confusion, and anxiety. It has been described that during the pre‐ictal period a series of alterations occur, which some being consider prodrome and other manifestations of seizures. There is a progressive neuronal activation before the seizure, responsible for the prodromal alterations related to behavioral and affective manifestations, reaching a climax that causes convulsive episodes in the brain, thus producing the epileptic seizure, and a return to zero after the seizure, thus explaining the patient's feeling of relief and improvement of psychiatric symptoms.^19^ The most frequent pre‐ictal manifestations reported as prodromes before were confusion, anxiety, irritability, and changes in mood,^29^ due to abnormal activations of brain regions such as the temporal and frontal lobes, coincident with the behavioral, and psychotic manifestations described as most prevalent in our findings.

In the ictal phase, although fewer case reports were reviewed, mood/anxiety symptoms, psychosis, and personality changes were the main psychiatric manifestations of seizures. Similarly, Wilson et al. observed that around 13% of these PWE experienced irritability and dysphoria associated with the ictal period.^37^ During seizures, epileptogenic discharges are generated that abnormally activate brain areas that could explain these manifestations. Such as “ictal panic” manifested in focal seizures of the mesial temporal lobe related to epileptogenic activity in temporal areas or frontotemporal limbic networks, including the amygdala.^38^ Or antisocial behavior and subclinical epileptic activity of ventromedial prefrontal networks. Also, psychotic manifestations were found in patients with temporal lobe epilepsy, in whom electroencephalographic discharges have been detected, in the septum and, with less intensity, in the structures of the medial temporal lobe, amygdala, and hippocampus. Hypoactivity of the frontal lobe also produces psychotic manifestations, explained by a phenomenon of “frontal‐temporal disconnection,” resulting in positive symptoms due to dysfunction of temporal structures.^39^

### 4.3 Bias and certainty in relation to the confidence of our findings

We found the certainty of evidence for our results from low to very low. The domains of inconsistency and imprecision were assessed as “serious”, because the heterogeneity and the confidence interval were not measured or reported in the included reviews. The absence of reviews in the topic calls for the development of high‐quality prospective primary studies, especially from specialized centers and the use of systematic psychiatric evaluations in the clinical evaluations of PWE. Regarding the risk of bias domain, it was also considered “serious” because the evaluation of the individual studies was not reported or between 25% and 50% were considered to be at high risk of bias. Appropriate design and development of systematic reviews following standardized guidelines is highly encouraged. In addition, future primary and secondary prevalence studies need to report their findings with confidence intervals using the appropriate method.^40^ Otherwise, our table of criteria for evaluating systematic reviews using the GRADE system can contribute to future umbrella reviews and be adapted depending on its objective.

### 4.4 Clinical applicability of our findings

The variety of psychiatric symptoms that present as manifestations of epilepsy could be misdiagnosed as PD. These situations would lead to diagnostic or therapeutic delays, and negative clinical outcomes. In a study of the prevalence of bipolar disorder in patients with epilepsy, it was found that in a group of 143 PWE, 11.9% were diagnosed with bipolar disorder; however, only 1.4% could be considered as a primary psychiatric disorder, because the manifestations of mania and hypomania would be epileptic manifestations.^41^ In the same line, in up to 13% of PWE, dysphoric disorders, with irritability and depressed mood, which manifested in interictal or pre‐ictal periods, generated difficulties for diagnosis and delayed treatment.^42^ Or the psychoses that occur in 4% of PWE in their postictal periods in focal epilepsy or with idiopathic generalized epilepsy.^43^ Therefore, it is important to perform a careful clinical assessment of patients to distinguish between peri‐ictal PM from “primary psychiatric manifestations” in people without epilepsy to optimize the treatment and prognosis.

There is a need for conducting research on PWE that have psychiatric manifestations such as during seizures, because these could predispose or increase the risk of developing psychiatric comorbidities. We found that emotional symptoms as seizure manifestations are frequent. Currently, there is no systematic approach in its evaluation and management and the treatment remains inadequate due in part to fear about the effect of psychiatric medications on seizure threshold, including some antidepressants and antipsychotics, and the lack of knowledge regarding the management of some psychoactive drugs.^12^ Additionally, there is a need for strengthening collaboration between neurologist and psychiatrist in the therapy of PWE.^44^

The presence of symptoms of anxiety, depression, and psychosis increases the difficulty of classifying them as primary psychiatric conditions or secondary to epilepsy. This situation has also been associated with a lack of adherence to medication for epilepsy and the worst disease trajectory, since the presence of psychiatric comorbidities has a negative impact on tolerance to antiseizure medication (ASM) with greater severity of adverse effects mainly in patients with severe depressive disorders. People with uncontrolled epilepsy may also suffer from depression or anxiety related to the fear of having new seizures, and due to the psychotropic effects of the combination therapy with ASM, thus forming a bidirectional relationship between both entities that increases challenges of management and impairing the quality of life of patients.^12^ Furthermore, comorbid psychiatric conditions are associated with an increased risk of suicide and premature death.^45^

### 4.5 Limitations

Our study has some limitations that should be considered when interpreting the results. Despite conducting a comprehensive search of the literature, there is a possibility that some studies might have been missing. However, it is unlikely that they were published outside of the included databases. Finally, our review relies on the quality of the systematic reviews included. This would be as well dependent on the quality of the primary observational studies. Selection and information bias, as well as the inherent biases introduced during the clinical diagnosis of epilepsy—mostly based on clinician experience and subjectivity.

## 5 CONCLUSIONS AND RECOMMENDATIONS

There is a significant proportion of PWE that present PM as part of the disease phenotype. In the pre‐ictal period, the common manifestations are confusion states, anxiety symptoms and irritability; in the ictal period, mood and anxiety symptoms, psychosis, and personality changes, and in the postictal period, mostly anxiety and depressive symptoms. It is recommended to maintain a high degree of suspicion of PM in PWE to prevent diagnostic and therapeutic errors and to carry out primary studies focused on the evaluation of the PM in PWE.

## AUTHOR CONTRIBUTIONS

CAD, MVA, KAM, ANF, and RRG conceptualized the study. ANF created the search strategy. ANF, RMR, and MCL performed the selection of the studies. MCL and RMR performed the extraction of the data. MCL and RMR assessed the quality analysis. MCL, ANF, RMR, and KPB contributed to the data gathering, cleaning, and visualization. CAD, JGB, and KPB supervised the processes. All authors wrote and approved the final version of the manuscript.

## CONFLICT OF INTEREST STATEMENT

None of the authors has any conflict of interest to disclose. We confirm that we have read the Journal‘s position on issues involved in ethical publication and affirm that this report is consistent with those guidelines.

## Supporting information


Appendix S1.

## Data Availability

The authors confirm that the data supporting the findings of this study are available within the article and its supplementary materials.
