# Supplementary material for: Peri‐ictal psychiatric manifestations in people with epilepsy: An umbrella review
Source: Epilepsia Open. 2024 May 30;9(4):1166–75. doi: 10.1002/epi4.12949 (PMC11296096; doi:10.1002/epi4.12949)
Supplement: Supplementary file 1 — Appendix S1. [file EPI4-9-1166-s001.docx]

**This is the supplementary material for**

**PERI-ICTAL PSYCHIATRIC MANIFESTATIONS IN PEOPLE WITH EPILEPSY: AN UMBRELLA REVIEW**

Authors: Carlos Alva-Diaz, Miguel Cabanillas-Lazo, Alba Navarro-Flores, Raisa N. Martinez-Rivera, Maria Valdeiglesias-Abarca, Krystel Acevedo-Marino, Kevin Pacheco-Barrios, Ramiro Ruiz-Garcia, Jorge Burneo.

**Contents**

[Supplementary Table 1. Search strategy 2](#_Toc139983123)

[Supplementary Table 2. Excluded studies 4](#_Toc139983124)

[Supplementary Table 3. Decision table based on GRADE system (Adaptation by authors). 8](#_Toc139983125)

[Supplementary Table 4. Overlap measurement 9](#_Toc139983126)

[Supplementary Figure 1. Quality of all included systematic reviews by AMSTAR-2 11](#_Toc139983127)

[Supplementary Table 5. Quality assessment of included systematic reviews (AMSTAR-2). 12](#_Toc139983128)

# **Supplementary Table 1. Search strategy**

| Database | Search strategy | Results |
| --- | --- | --- |
| Pubmed | #1: Psychosis[tiab] or “mood disorders”[tiab] or “mood disorder”[tiab] or anxiety[tiab] or “personality disorders”[tiab] or suicide[tiab] or “postictal psychosis”[tiab] or “depression”[tiab] or mania[tiab] or neurosis[tiab] or aura[tiab]  #2: Epilepsy[tiab] or seizure[tiab] or “Gelastic Epilepsy”[tiab] or Epilepsy Frontal Lobe[mesh] or Epilepsy Frontal Lobe[tiab] or epilepsy temporal lobe[mesh] or “epilepsy temporal lobe”[tiab] or “hippocampal sclerosis”[tiab] or (hippocampus[Mesh] and sclerosis[mesh])  #3: ("Systematic Review"[Publication Type] OR "Meta-Analysis"[Publication Type] OR "Meta-Analysis as Topic"[Mesh] OR "Systematic Review"[TIAB] OR “Meta Analysis”[TIAB] OR Metanalysis[TIAB] OR Metaanalysis[TIAB])  Search strategy: #1 AND #2 AND #3 | 467 |
| Scopus | #1 TITLE-ABS-KEY( epileps* OR &quot;Seizure Disorder*&quot; )  #2 (TITLE-ABS-KEY(disorder*) and TITLE-ABS-  KEY(pyscho* or Schizoaffective or mood or affective or mental  or behavior or anxiety or personality or somatoform or neuroticor  dissociative or bipolar or disruptive or Psychiatri*)) or (TITLE-  ABS-KEY(disease*) and TITLE-ABS-KEY(psychiatric or  mental)) or TITLE-ABS-KEY(anxiety or neurosis)  #3 TITLE-ABS(&quot;Systematic Review&quot; or &quot;Meta-Analysis&quot; or  Metanalysis)  (#1 AND #2) AND #3 | 530 |
| PsycInfo | #1 (epileps* or Seizure).ab,kw,mh,sh,ti,tx.  #2 &quot;disorder*&quot;.ab,kw,mh,sh,ti,tx.  #3 (pyscho* or Schizoaffective or mood or affective or mentalor  behavior or anxiety or personality or somatoform or neuroticor  dissociative or bipolar or disruptive or  psychiatri*).ab,kw,mh,sh,ti,tx.  #4 &quot;disease*&quot;.ab,kw,mh,sh,ti,tx.  #5 (psychiatric or mental).ab,kw,mh,sh,ti,tx.  #6 #2 AND #3  #7 #4 AND #5  #8 #6 OR #7  #9 (anxiety or neurosis).ab,kw,mh,sh,ti,tx.  #10 #8 OR #9  #11 #1 AND #10  #12 (&quot;Systematic Review&quot; or &quot;Meta-Analysis&quot; or  Metanalysis).ab,kw,mh,sh,ti,tx.  #13 #11 AND #12 | 437 |
| Web of Science | #1 TS=( epileps* OR Seizure)  #2 (TS=(disorder*) and TS=(pyscho* or Schizoaffective or mood  or affective or mentalor behavior or anxiety or personality or  somatoform or neuroticor dissociative or bipolar or disruptive or  psychiatri*)) or (TS=( disease*) and TS=(psychiatric or mental))  or TS=(anxiety or neurosis)  #3 TS=(&quot;Systematic Review&quot; or &quot;Meta-Analysis&quot; or Metanalysis)  (#1 AND #2) AND #3 | 411 |
| Epistemonikos | (title:(title:(epileps* OR Seizure) AND (title:(psychosis OR  “disorder psychotic” OR “disorders psychotic” OR psychoses OR “mood disorders” OR “disorder mood” OR “affective disorder” OR depression OR mania OR anxiety OR neurosis OR “personality disorders” OR “personality disorder” OR psychiatric OR Schizoaffective OR somatoform OR disruptive OR psychiatri*) OR abstract:(psychosis OR “disorder psychotic” OR “disorders psychotic” OR psychoses OR “mood disorders” OR “disorder mood” OR “affective disorder” OR depression OR mania OR anxiety OR neurosis OR “personality disorders” OR “personality disorder” OR psychiatric OR Schizoaffective OR somatoform OR disruptive OR psychiatri*))) OR abstract:(title:(epileps* OR Seizure) AND (title:(psychosis OR “disorder psychotic” OR “disorders psychotic” OR psychoses OR “mood disorders” OR “disorder mood” OR “affective disorder” OR depression OR mania OR anxiety OR neurosis OR “personality disorders” OR “personality disorder” OR psychiatric  OR Schizoaffective OR somatoform OR disruptive OR  psychiatri*) OR abstract:(psychosis OR “disorder psychotic” OR “disorders psychotic” OR psychoses OR “mood disorders” OR “disorder mood” OR “affective disorder” OR depression OR mania OR anxiety OR neurosis OR “personality disorders” OR “personality disorder” OR psychiatric OR Schizoaffective OR somatoform OR disruptive OR psychiatri*))))  Last 5 years  Publication type: Systematic Review | 148 |

# **Supplementary Table 2. Excluded studies**

| Author-year | Title | Exclusion reasons |
| --- | --- | --- |
| Randhawa, J. (2019) | Affect-induced reflex seizures (AIRS): A case series based on a systematic literature review | NO SR |
| Scott, A. J (2017) | Anxiety and depressive disorders in people with epilepsy: A meta-analysis | PSYQUIATRIC COMORBID |
| Yang, T. W. (2020) | Anxiety disorders in outpatient clinics of epilepsy in tertiary care hospitals: A meta-analysis | PSYQUIATRIC COMORBID |
| Pinquart, M. (2011) | Anxiety in children and adolescents with chronic physical illnesses: a meta-analysis | PSYQUIATRIC COMORBID |
| Hogberg, G. (2011) | Anxiety in chronic illness: General salutogenic and specific pathological factors | NO SR |
| Beyenburg, S.  (2005) | Anxiety in patients with epilepsy: systematic review and suggestions for clinical management | NO SR |
| Pinquart, M. (2011) | Behavior problems in children and adolescents with chronic physical illness: a meta-analysis | NOT POPULATION |
| Da Costa, C. (2017) | Clinical and neuropsychological evaluation of attention in children and adolescents with epilepsy: A systematic review | NO SR |
| Helmstaedter, C. (2013) | Cognitive outcomes of different surgical approaches in temporal lobe epilepsy | NO SR |
| Walsh, S.  (2018) | Comorbid depression and associated factors in PNES versus epilepsy: Systematic review and meta-analysis | NOT POPULATION |
| Muhigwa, A.  (2020) | Comorbidities of epilepsy in low and middle-income countries: systematic review and meta-analysis | PSYQUIATRIC COMORBID |
| Sequeira, A.  (2019) | A Comparison Among the Prevalence of Alexithymia in Patients With Psychogenic Nonepileptic Seizures, Epilepsy, and the Healthy Population: A Systematic Review of the Literature | PSYQUIATRIC COMORBID |
| Dessie, G.  (2019) | Depression among epileptic patients and its association with drug therapy in sub-Saharan Africa: A systematic review and meta-analysis | PSYQUIATRIC COMORBID |
| Forthoffer, N  (2020) | Depression could modulate neuropsychological status in epilepsy | NO SR |
| Fiest, K. M.  (2013) | Depression in epilepsy | PSYQUIATRIC COMORBID |
| Fiest, K. M  (2013) | Depression in epilepsy A systematic review and meta-analysis | duplicated |
| Gill, S. J.  (2017) | Depression screening tools in persons with epilepsy: A systematic review of validated tools | NOT OUTCOMES |
| Pinquart, M.  (2011) | Depressive symptoms in children and adolescents with chronic physical illness: An updated meta-analysis | NOT POPULATION |
| Hellmann-Regen, J.  (2013) | Depressive syndromes in neurological disorders | NO SR |
| Reilly, C.  (2019) | Do behavior and emotions improve after pediatric epilepsy surgery? A systematic review | NOT OUTCOMES |
| De Barros L.  (2020) | Electroencephalogram alterations associated with psychiatric disorders in temporal lobe epilepsy with mesial sclerosis: A systematic review | NOT POPULATION |
| Repchak, A.  (2016) | Epileptic Catatonia: A Case Series and Systematic Review | NO SR |
| Hrabok, M.  (2013) | An evidence-based checklist to assess neuropsychological outcomes of epilepsy surgery: how good is the evidence? | NO SR |
| Pericall, M.  (2017) | FACTORS ASSOCIATED WITH PSYCHIATRIC DISORDER IN CHILDREN WITH EPILEPSY (CWE): A SYSTEMATIC REVIEW | ABSTRACT |
| Kwon, O.  (2017) | Fatigue in epilepsy: A systematic review and meta-analysis | NOT OUTCOMES |
|  | Frontal Lobe Epilepsy: A Primer for Psychiatrists and a Systematic Review of Psychiatric Manifestations | DUPLICATED |
| Lax-Pericall,M.  (2019) | Gender and psychiatric disorders in children with epilepsy. A meta-analysis | NO SR OF PREVALENCE |
| Farooq, S.  (2015) | Interventions for psychotic symptoms concomitant with epilepsy | NO SR OF PREVALENCE |
| Ramanujam, B.  (2017) | Is Depression Related to Low Folate Levels in People with Epilepsy? An Observational Study and Meta-analysis | NO SR |
| Kim, M.  (2018) | Major depressive disorder in epilepsy clinics: A meta-analysis | NOT OUTCOMES |
| Abraham, N.  (2019) | A Meta-Analysis of the Rates of Suicide Ideation, Attempts and Deaths in People with Epilepsy | NOT OUTCOMES |
| Yrondi, A.  (2017) | [Mood disorders and epilepsy surgery: A review] | NOT OUTCOMES |
| Robertson, J.  (2015) | Mortality in people with intellectual disabilities and epilepsy: A systematic review | NOT POPULATION |
| Fazel, S.  (2009) | Neurological disorders and violence: A systematic review and meta-analysis with a focus on epilepsy and traumatic brain injury | NO SR OF PREVALENCE |
| Sherman, E.  (2011) | Neuropsychological outcomes after epilepsy surgery: systematic review and pooled estimates | NO SR OF PREVALENCE |
| Flint, A.  (2017) | Neuropsychological outcomes following paediatric temporal lobe surgery for epilepsies: Evidence from a systematic review | NOT OUTCOMES |
| Verche E.  (2018) | Neuropsychology of frontal lobe epilepsy in children and adults: Systematic review and meta-analysis | NO SR OF PREVALENCE |
| Kim, D.  (2019) | Optimal cutoff score of the Neurological Disorders Depression Inventory for Epilepsy (NDDI-E) for detecting major depressive disorder: A meta-analysis | NOT OUTCOMES |
| Johnson, A.  (2018) | Panic and epilepsy in adults: A systematic review | NO SR OF PREVALENCE |
| Weyand, C.  (2016) | Pediatric Psychology and Epilepsy: A State of the Field and Call to Action | NO SR |
| Cleary, R.  (2013) | Predicting and preventing psychopathology following temporal lobe epilepsy surgery | NOT OUTCOMES |
| Fond, G.  (2019) | [Prevalence of major depression in France in the general population and in specific populations from 2000 to 2018: A systematic review of the literature] | NOT POPULATION |
| Dagar, A.  (2020) | Psychiatric Comorbidities in Pediatric Epilepsy | NOT OUTCOMES |
| Killeen, Z.  (2017) | Psychiatric comorbidity with hypothalamic hamartoma: Systematic review for predictive clinical features | NOT OUTCOMES |
| Macrodimitris, S.  (2011) | Psychiatric outcomes of epilepsy surgery: a systematic review | NOT OUTCOMES |
| Pinquart, M.  (2017) | Psychological health of children with chronic physical illness and their parents - results from meta-analyses. | NO SR |
| Bennett S.  (2015) | Psychological interventions for mental health disorders in children with chronic physical illness: A systematic review | SR OF INTERVENTION |
| Rodenburg, R.  (2005) | Psychopathology in children with epilepsy: a meta-analysis | NOT OUTCOMES |
| Quintas, R.  (2012) | Psychosocial difficulties in people with epilepsy: a systematic review of literature from 2005 until 2010 | NOT OUTCOMES |
| Gandy, M.  (2012) | Psychosocial predictors of depression and anxiety in patients with epilepsy: a systematic review | NOT OUTCOMES |
| Monteagudo-Gimeno, E.  (2008) | Relationship between cognition and psychopathology in drug-resistant epilepsy: A systematic review | NOT OUTCOMES |
| Secinti, E.  (2017) | Research Review: Childhood chronic physical illness and adult emotional health â€“ a systematic review and meta-analysis | NOT POPULATION |
| Reilly, C. (2013) | Review: Psychopathology in childhood epilepsy | NOT OUTCOMES |
| Lacey, C. (2015) | Risk factors for depression in community-treated epilepsy: systematic review | NOT OUTCOMES |
| Yang, Y. (2020) | Risk factors for depression in patients with epilepsy: A meta-analysis | NOT OUTCOMES |
| Ferro,M. (2014) | Risk factors for health-related quality of life in children with epilepsy: A meta-analysis | NO SR OF PREVALENCE |
| Irwin, L. (2014) | Risk factors for psychosis secondary to temporal lobe epilepsy: a systematic review. | NO SR OF PREVALENCE |
| Bora, E. (2016) | Social cognition in temporal lobe epilepsy: A systematic review and meta-analysis | NOT OUTCOMES |
| Kok, T. (2014) | Social Competence in Children with Brain Disorders: A Meta-analytic Review | NOT POPULATION |
| Puka, K. (2019) | Social outcomes for adults with a history of childhood-onset epilepsy: A systematic review and meta-analysis | NOT OUTCOMES |
| Redden, L. (2011) | Suicidality and divalproex sodium: analysis of controlled studies in multiple indications | SR OF INTERVENTION |
| Pereira, A. (2013) | Suicidality associated with antiepileptic drugs: Implications for the treatment of neuropathic pain and fibromyalgia | NOT POPULATION |
| Bell, G. (2009) | Suicidality in people taking antiepileptic drugs: What is the evidence? | NO SR |
| Harris, E. (1997) | Suicide as an outcome for mental disorders. A meta-analysis | NOT POPULATION |
| Bell, G. (2009) | Suicide in people with epilepsy: how great is the risk? | NOT OUTCOMES |
| Lu, E. (2021) | Systematic literature review on psychiatric comorbidities in patients with epilepsy | PSYQUIATRIC COMORBID |
| Fay-McClymont, T. (2012) | Systematic review and case series of neuropsychological functioning after epilepsy surgery in children with dysembryoplastic neuroepithelial tumors (DNET) | PSYQUIATRIC COMORBID |
| Kolc, K. (2019) | A systematic review and meta-analysis of 271 PCDH19-variant individuals identifies psychiatric comorbidities, and association of seizure onset and disease severity | SR OF INTERVENTION |
| Scott, A. (2020) | Systematic Review and Meta-Analysis of Anxiety and Depression in Youth With Epilepsy | PSYQUIATRIC COMORBID |
| Vanool, J. (2016) | A systematic review of neuropsychiatric comorbidities in patients with both epilepsy and intellectual disability | PSYQUIATRIC COMORBID |
| Loughman, A. (2016) | A Systematic Review of Psychiatric and Psychosocial Comorbidities of Genetic Generalised Epilepsies (GGE) | PSYQUIATRIC COMORBID |
| Allebone, J. (2018) | Systematic review of structural and functional brain alterations in psychosis of epilepsy | NO SR OF PREVALENCE |
| Baroni, G. (2016) | Variables associated with co-existing epileptic and psychogenic nonepileptic seizures: a systematic review | NO SR OF PREVALENCE |
| Wang, H. (2023) | Suicidality and epilepsy: A systematic review and meta-analysis | PSYQUIATRIC COMORBID |
| Tsigebrhan, R. (2023) | Co-morbid mental health conditions in people with epilepsy and association with quality of life in low- and middle-income countries: a systematic review and meta-analysis | PSYQUIATRIC COMORBID |
| Qin, S. (2022) | Exploring the association between epilepsy and depression: A systematic review and meta-analysis | PSYQUIATRIC COMORBID |
| Hue, C. (2022) | Depression and suicide after temporal lobe epilepsy surgery: A systematic review | NOT OUTCOMES |
| Chu, C (2022) | Association between epilepsy and risk of depression: A meta-analysis. | NO SR OF PREVALENCE |
| Gurgu, R. (2023) | Psychiatric comorbidities in adult patients with epilepsy | PSYQUIATRIC COMORBID |

- *SR: systematic review

| **Start with** | **Criteria** | **Risk of bias** | **Inconsistency** | **Indirectness** | **Imprecision** | **Publication bias** | | |
| --- | --- | --- | --- | --- | --- | --- | --- | --- |
| High confidence:  Studies included were cross-sectional with the main objective of measuring prevalence | no serious | <25% of studies included in meta-analysis have low risk of bias | I^2^ <30% | The inclusion criteria correspond to the Population of the umbrella review. | Adequate sample size (>300 participants) **AND** narrow confidence interval | No graphed, estimated, or methodological publication bias | | |
| Low confidence: Included cross-sectional studies without the main objective of measuring prevalence | Serious | No risk of bias analysis of the included studies is performed **OR** >25% and <50% of studies included in meta-analysis have low risk of bias | 30-60% **OR** no detail | The inclusion criteria do not correspond to the Population of the umbrella review | Non-adequate sample size (≤ 300 participants) **OR** no confidence interval presented | Asymmetry evaluation in the Funnel Plot. Using publication bias statistics (Egger) | Analyze if the search was exhaustive |  |
|  | Very serious | >50% of studies included in meta-analysis have low risk of bias | I^2^ >60% |  |  |  |  |  |

# **Supplementary Table 3. Decision table based on GRADE system (Adaptation by authors).**

# **Supplementary Table 4. Overlap measurement**

| **Systematic Review/ Author** | **Corbet et al.** | **Besag et al.** | **Subota et al.** |
| --- | --- | --- | --- |
| Ng (2006) |  |  |  |
| Procaccini (2006) |  |  |  |
| Valdueza (1994 ) |  |  |  |
| Fornazzari et al, (1992) |  |  |  |
| Abla (2010) |  |  |  |
| Akiyama et al, (2015) |  |  |  |
| Alkan (2009) |  |  |  |
| Alving and Beniczky (2013) |  |  |  |
| Andrew (2008) |  |  |  |
| Arita (1999) |  |  |  |
| Arzimanoglou (2003) |  |  |  |
| Bauer et al, (2005) |  |  |  |
| Berkovic (1988) |  |  |  |
| Bestha and Arora, (2009) |  |  |  |
| BourionBédès et al (,2013) |  |  |  |
| Brandberg (2003) |  |  |  |
| Castano de la Mota (2011) |  |  |  |
| Cercy & Kuluva,(2009) |  |  |  |
| Chicharro Ciuffardi et al, (2012) |  |  |  |
| Coffey, (2013) |  |  |  |
| Cukier (2013) |  |  |  |
| Cull et al (1996) |  |  |  |
| Delalande (2003) |  |  |  |
| Eisenshenk et al, (2014) |  |  |  |
| Elmi et al,( 2011) |  |  |  |
| Fohlen et al,( 2004) |  |  |  |
| Frattali (2001) |  |  |  |
| Giuccioli et al. (1990) |  |  |  |
| Hughes et al. (1993) |  |  |  |
| Janz et al. (1969) |  |  |  |
| Jayalakshmi (2014) |  |  |  |
| Kameyana (2009) |  |  |  |
| Kaplan & Stagg, (2011) |  |  |  |
| Kovac et al, (2009) |  |  |  |
| Kuzniecky (1997) |  |  |  |
| La Vega Talbot et al, (2006) |  |  |  |
| Leal (2002 & 2003) |  |  |  |
| Lee and No (2005) |  |  |  |
| Leentjens & Pepplinkhuize,( 1998) |  |  |  |
| Liguori et al, (2014) |  |  |  |
| Lopez et al, (2010) |  |  |  |
| Luat et al, (2008) |  |  |  |
| Manfioli et al, (2013) |  |  |  |
| Mottolese (2001) |  |  |  |
| Murphy (2000) |  |  |  |
| Neilson et al, (2014) |  |  |  |
| Ng (2011) |  |  |  |
| Nguyen (2003) |  |  |  |
| Nishida et al, (2005) |  |  |  |
| Ozyurt et al, (2015) |  |  |  |
| Palmini( 2002) |  |  |  |
| Pascualy et al, (1997) |  |  |  |
| Petitmengin et al. (2006) |  |  |  |
| Pinikahana and Dono (2009) |  |  |  |
| Pinikahana and Dono (2009) |  |  |  |
| Powell et al,(1997) |  |  |  |
| Rajna et al. (1997) |  |  |  |
| Ramsey, (1999) |  |  |  |
| Riggio,(2009) |  |  |  |
| Rosenfeld ( 2001) |  |  |  |
| Scaramelli et al. (2009) |  |  |  |
| Schulze-Bonhage (2004) |  |  |  |
| Schulze-Bonhage et al. (2006) |  |  |  |
| Sebit & Suleman,(1998) |  |  |  |
| Shih et al, (2009) |  |  |  |
| Sinclair & Snyder, (2008) |  |  |  |
| Southwell (2018 ) |  |  |  |
| Specchio (2015) |  |  |  |
| Sue et al. (1986) |  |  |  |
| Takaya et al, (2005) |  |  |  |
| Thomas et al,(1999) |  |  |  |
| Trebuchon et al, (2013) |  |  |  |
| Unnwongse et al, (2010 |  |  |  |
| Veendrick-Meekes ( 2007) |  |  |  |
| Weissenberger ( 2001) |  |  |  |
| Yang-Je Cho, et al,(2009) |  |  |  |

# **Supplementary Figure 1.** Quality of all included systematic reviews by AMSTAR-2


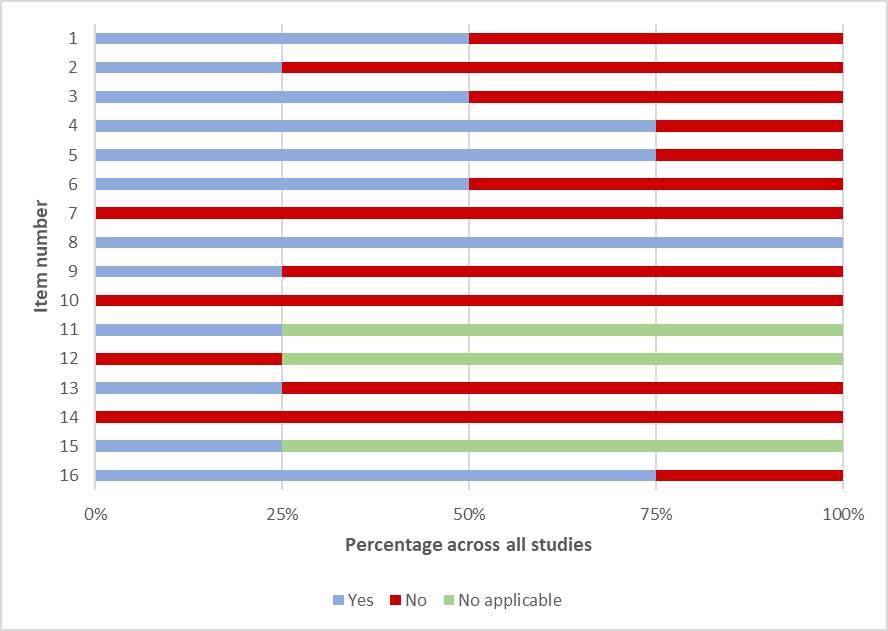


# **Supplementary Table 5. Quality assessment of included systematic reviews (AMSTAR-2).**

| AMSTAR-2 Question | Corbet (2019) | Besag (2018) | Subota (2019) | Gold (2016) |
| --- | --- | --- | --- | --- |
| 1. Did the research questions and inclusion criteria for the review include the components of PICO? | Yes | No | No | Yes |
| 1. Did the report of the review contain an explicit statement that the review methods were established prior to the conduct of the review and did the report justify any significant deviations from the protocol? | No | No | Yes | No |
| 1. Did the review authors explain their selection of the study designs for inclusion in the review? | Yes | No | Yes | No |
| 1. Did the review authors use a comprehensive literature search strategy? | Yes | No | Yes | Yes |
| 1. Did the review authors perform study selection in duplicate? | Yes | No | Yes | Yes |
| 1. Did the review authors perform data extraction in duplicate? | Yes | No | Yes | No |
| 1. Did the review authors provide a list of excluded studies and justify the exclusions? | No | No | No | No |
| 1. Did the review authors describe the included studies in adequate detail? | Yes | Yes | Yes | Yes |
| 1. Did the review authors use a satisfactory technique for assessing the risk of bias (RoB) in individual studies that were included in the review? | No | No | Yes | No |
| 1. Did the review authors report on the sources of funding for the studies included in the review? | No | No | No | No |
| 1. If meta-analysis was performed did the review authors use appropriate methods for statistical combination of results? | NA | NA | Yes | NA |
| 1. If meta-analysis was performed, did the review authors assess the potential impact of RoB in individual studies on the results of the meta-analysis or other evidence synthesis? | NA | NA | No | NA |
| 1. Did the review authors account for RoB in individual studies when interpreting/ discussing the results of the review? | No | No | Yes | No |
| 1. Did the review authors provide a satisfactory explanation for, and discussion of, any heterogeneity observed in the results of the review? | No | No | No | No |
| 1. If they performed quantitative synthesis did the review authors carry out an adequate investigation of publication bias (small study bias) and discuss its likely impact on the results of the review? | NA | NA | Yes | NA |
| 1. Did the review authors report any potential sources of conflict of interest, including any funding they received for conducting the review? | Yes | Yes | Yes | No |
| Score | 7/13 | 2/13 | 11/16 | 4/13 |
